# Signal Variability and Cognitive Function in Older Long-Term Survivors of Breast Cancer with Exposure to Chemotherapy: A Prospective Longitudinal Resting-State fMRI Study

**DOI:** 10.3390/brainsci12101283

**Published:** 2022-09-23

**Authors:** Bihong T. Chen, Zikuan Chen, Frank Deng, Sunita K. Patel, Mina S. Sedrak, James C. Root, Tim A. Ahles, Marianne Razavi, Heeyoung Kim, Can-Lan Sun, William Dale

**Affiliations:** 1 Department of Diagnostic Radiology, City of Hope National Medical Center, 1500 East Duarte Road, Duarte, CA 91010, USA; 2 Center for Cancer and Aging, City of Hope National Medical Center, Duarte, CA 91010, USA; 3 Department of Population Science, City of Hope National Medical Center, Duarte, CA 91010, USA; 4 Department of Medical Oncology, City of Hope National Medical Center, Duarte, CA 91010, USA; 5 Neurocognitive Research Lab, Memorial Sloan Kettering Cancer Center, New York, NY 10065, USA; 6 Department of Supportive Care Medicine, City of Hope National Medical Center, Duarte, CA 91010, USA

**Keywords:** breast cancer, cancer-related cognitive impairment, chemotherapy, blood-oxygen-level-dependent (BOLD) signal variability, resting-state fMRI

## Abstract

The purpose of this study was to assess the effect of chemotherapy on brain functional resting-state signal variability and cognitive function in older long-term survivors of breast cancer. This prospective longitudinal study enrolled women age ≥ 65 years of age who were breast cancer survivors after exposure to chemotherapy (CH), age-matched survivors not exposed to chemotherapy, and healthy controls. Participants completed resting-state functional brain MRI and neurocognitive testing upon enrollment (timepoint 1, TP1) and again two years later (timepoint 2, TP2). There were 20 participants in each of the three groups at TP1. The CH group showed a significant decrease in SD_BOLD_ (blood-oxygen-level-dependent signal variability in standard deviation) in the right middle occipital gyrus (ΔSD_BOLD_ = −0.0018, *p* = 0.0085, q (p_FDR_) = 0.043 at MNI (42, −76, 17)) and right middle temporal gyrus (ΔSD_BOLD_ = −0.0021, *p* = 0.0006, q (p_FDR_) = 0.001 at MNI (63, −39, −12)). There were negative correlations between the crystallized composite scores and SD_BOLD_ values at the right inferior occipital gyrus (correlation coefficient r = −0.84, *p* = 0.001, q (p_FDR_) = 0.016) and right middle temporal gyrus (r = −0.88, *p* = 0.000, q (p_FDR_) = 0.017) for the CH group at TP1. SD_BOLD_ could be a potentially useful neuroimaging marker for older long-term survivors of breast cancer with exposure to chemotherapy.

## 1. Introduction

Chemotherapy is an effective treatment for cancer, but it can negatively impact cognitive function in cancer survivors [1]. Older adults are at an increased risk for neurotoxicity from chemotherapy [2,3,4]. The association between chemotherapy and cancer-related cognitive impairment (CRCI) has been reported in long-term survivors. Prior research has shown that breast cancer survivors performed worse than healthy controls in cognitive testing more than 20 years after chemotherapy [5]. Another study found that chemotherapy was associated with poorer self-reported cognitive function in a large cohort of postmenopausal women with breast cancer [6]. Nevertheless, the biological mechanism underlying CRCI remains poorly understood, particularly in older adults, who are at high risk of suffering from cancer.

Magnetic resonance imaging (MRI) has shown brain alterations and CRCI in breast cancer survivors [7]. Moreover, there are measurable effects of aging, cancer, and chemotherapy on brain structure and function, lending support to using neuroimaging to evaluate CRCI [8,9]. Reduced gray matter, disorganized white matter, and diminished cerebral responsiveness have been found in long-term breast cancer survivors with a history of chemotherapy [7]. Miao et al. showed chemotherapy-associated reduction of functional connectivity in the anterior cingulate cortex using resting-state brain functional MRI (rs-fMRI) [10]. Our own fMRI study of older women with breast cancer also showed a weaker functional network connectivity shortly after chemotherapy [11]. A fMRI study in younger patients aged 52.9 ± 8.6 years demonstrated increased activation in the posterior middle temporal gyrus of patients with breast cancer after chemotherapy, largely attributed to compensatory effects [12]. Nevertheless, more work is needed to identify the neural correlates underlying CRCI, which may help us assess the trajectory of CRCI and aging in older cancer survivors.

A timeseries of spatiotemporal images composed of voxel-wise brain blood-oxygen-level-dependent (BOLD) signals is acquired in a rs-fMRI [13,14]. BOLD signals can be processed for functional connectivity (FC) mapping, such as default mode network (DMN) for inter-signal correlations, or for mapping of local regional signal variability for intra-signal analysis. The standard deviation (SD) of BOLD signal variability through intra-signal analysis, i.e., SD_BOLD_, is a sensitive measure for mathematically characterizing brain functional status [15,16,17]. Although FC and SD measures are different yet complimentary features for brain functional characterization, SD_BOLD_ has been shown to be a more sensitive parameter for detecting subtle brain changes in aging studies, and it is therefore more appropriate for characterization of the CRCI in long-term older cancer survivors. Garrett et al. showed that SD_BOLD_ was coupled with dynamic functional integration and cognitive performance in aging [18]. They found that the SD_BOLD_ trajectory followed an inverted U-shape across the lifespan, i.e., from low variability in infancy, to high variability in young adulthood to lower variability in older adulthood [19]. Another rs-fMRI study reported that the signal variability could reflect regional neural changes [20]. These studies have shown that SD_BOLD_ being capable of mapping local regional signal variability may be a candidate measure for assessing subtle brain alterations seen in aging and CRCI.

In this study, we report results from a prospective longitudinal rs-fMRI study of older long-term survivors of breast cancer with exposure to chemotherapy (CH) as compared to age-matched older women with breast cancer but no chemotherapy (NC) and healthy controls (HC). We hypothesized that older survivors of breast cancer who received adjuvant chemotherapy would exhibit alterations in SD_BOLD_ on rs-fMRI and the SD_BOLD_ alterations would be correlated with neurocognitive testing scores.

## 2. Materials and Methods

### 2.1. Study Design

This was a neuroimaging sub study of a multi-center clinical trial (parent trial: Cognition in Older Breast Cancer Survivors: Treatment Exposure, APOE, and Smoking History, NCT02122107). This observational study enrolled women aged 65 years or older who were long-term (from 5 to 15 years) breast cancer survivors (Stage I–III) exposed to chemotherapy (CH), long-term breast cancer survivors (Stage I–III) not exposed to chemotherapy (NC), and healthy control participants (HC) matched by age. Participants with a history of neurological, psychiatric, neurodegenerative, or cerebrovascular disease were excluded. We screened potential participants using two eligibility criteria checklists both prior to initial enrollment and at the follow-up assessment (see Supplementary files for the checklists). The first seven-item eligibility checklist was for the parent study, which included questions for antidepressant or antianxiety medication, history of stroke or head injury, cancer diagnosis, diagnosis of a major Axis I psychiatric disorder, or any visual or auditory impairment that would preclude ability to complete assessments. This neuroimaging sub study enrolled participants who were already enrolled for the parent study. The second three-item criteria were specifically for this neuroimaging sub study including questions for their enrollment status in the parent study, their handedness, and MRI safety check with questions for claustrophobia, cardiac pacer, and or orbital metal implant.

All participants completed rs-fMRI and neurocognitive assessments using the NIH (National Institute of Health) Toolbox Cognition Battery [21] upon enrollment (time point 1, TP1) and again two years later (time point 2, TP2). The participants’ clinical and demographic information was abstracted from their medical records and enrollment questionnaire (Table 1). We obtained written informed consent from all participants. This study was approved by our Institutional Review Board and was conducted in accordance with institutional guidelines and the Declaration of Helsinki.

### 2.2. Rs-fMRI Acquisition and Analysis

The rs-fMRI data was obtained on the same 3T Verio Siemens scanner (Siemens, Erlangen, Germany) using a standard gradient-recalled echo-planar sequence with the parameter setting: twelve-channel head coil, TR/TE = 2000/25 milliseconds, voxel = 3.5 × 3.5 × 3.5 mm^3^, flip angle = 80°, matrix = 64 × 64 × 32, total volume number = 160, and total acquisition time = 5.4 min. The participants were instructed to close their eyes but stay awake without thinking of anything during the scan. The study neuroradiologist (BTC) evaluated all images to rule out incidental brain pathology.

Each raw rs-fMRI dataset was made of a spatiotemporal time series of volumes in a 4D array and was preprocessed with Statistical Parametric Mapping (SPM 12). Preprocessing included image alignment, normalization to the Montreal Neurological Institute (MNI) space with voxels resampled in 3 × 3 × 3 mm^3^, and subsequent spatial smoothing with a Gaussian kernel of full-width at half-maximum (FWHM) = 6 mm (≤2 voxels) [22]. We removed the first four time points to ensure the stability of the imaging data. Each subject-specific rs-fMRI dataset was represented by a 4D array with a size of 53 × 63 × 46 × 156 for both TP1 and TP2 after preprocessing.

From the preprocessed rs-fMRI data in a 4D matrix, we extracted the voxel-wise timeseries signals (i.e., a 1D series of 156 timepoints at each voxel), performed detrending and bandpass filtering in a passband = [0.01, 0.15] Hz, and extracted 1D voxel-wise signals from which we calculated its SD_BOLD_ over all voxels in the whole-brain space. Subsequently, we obtained a 3D SD_BOLD_ map in a 53 × 63 × 46 matrix for each 4D rs-fMRI dataset. A representative SD_BOLD_ extraction showing two voxel-wise timeseries signals extracted from the same voxel at TP1 and TP2 is presented in Figure S1.

De Ruiter et al. have reported significant alterations of BOLD signal over the whole brain space in their fMRI studies [7,23], which included 24 significant MNI (Montreal Neurological Institute) coordinates in the regions of bilateral dorsolateral prefrontal cortex, precuneus, lateral posterior parietal cortex, premotor cortex, dorsal stratum, occipital cortex, and inferior temporal gyrus. We adopted their 24 significant MNI coordinates (x, y, z) from their reports as regions of interest (ROIs) for our regional BOLD signal variability analysis [7,23]. Multiple comparison analysis with a false discovery rate (FDR) correction was performed in the local ROI regions around these 24 significant coordinates. Each local region was defined as a neighborhood of the size of 5 × 5 × 5 = 125 voxels for each of the 24 MNI coordinates. It was assumed that brain functional activity exerted spatial continuity and contingency in the neighborhood of an identified voxel. All voxel-wise t-test *p*-values in the local ROIs were analyzed with the FDR algorithm for a small volume correction to obtain the corrected *p* values after multiple comparison, denoted by q-value (q = p_FDR_).

### 2.3. Neurocognitive Testing

All participants underwent neurocognitive testing with the computerized NIH Toolbox Cognition Battery at both TP1 and TP2 in a room outside the MRI scanner [21]. This battery generated seven individual scores for various cognitive functions including memory, executive function, processing speed, and language, and three composite scores, including the fluid composite score, total composite score, and crystallized composite score. The seven individual scores from the NIH Toolbox cognition battery tested the following cognitive functions: picture vocabulary test for language comprehension, oral reading recognition test for language-reading decoding, picture sequence memory test for episodic memory, list sorting working memory test for working memory, pattern comparison processing speed test for processing speed, Flanker inhibitory control and attention test for executive function-inhibitory control and attention, and dimensional change card sort test for executive function.

### 2.4. Statistical Analysis

The clinical and demographic data were evaluated using analysis of variance (ANOVA) for continuous variables, and chi-square or Fisher’s exact tests for categorical variables. The linear mixed models were conducted with compound symmetry covariance to examine the cognitive testing data at TP1 and the changes over time, accounting for within-subject correlations in repeated measures [24]. The following terms were included in the models: group, time, and group × time.

For each subject’s brain imaging data, we obtained one pair of brain SD_BOLD_ matrices (SD_BOLD_[TP1], SD_BOLD_[TP2]) in a 3D matrix for TP1 and TP2. The SD_BOLD_ changes between two time points (TP1, TP2) for the three groups (CH, NC, HC), as well as the group differences of the SD_BOLD_ changes, were assessed using a mixed-design repeated measurement ANOVA model in SPM 12. We conducted paired two-sample t-tests for within-group change for each group by calculating the longitudinal SD_BOLD_ dataset, as denoted by ΔSD_BOLD_ = SD_BOLD_(TP2) − SD_BOLD_(TP1), with which ΔSD_BOLD_ (x, y, z) was interpreted as the longitudinal change at brain voxel coordinate (x, y, z) as determined by paired t-test. We evaluated the between-group difference at TP1 and the group × time interaction by unpaired two-sample t-tests. The group x time interaction was performed for intergroup comparison between two within-group longitudinal changes using unpaired two-sampled t-tests on voxel-wise comparisons. The statistical significance was set at q (p_FDR_) < 0.05 after FDR correction for multiple comparisons.

Correlative analysis was performed using a ROI approach by computing pair-wise Pearson correlation coefficients between the SD_BOLD_ values of the brain regions showing significant reduction in the CH group and the three composite scores from the NIH Toolbox Cognition Battery (Table 2). We performed the correlation with the ROI approach rather than using a whole-brain matrix. This was because of the understanding that these significant brain regions implicated their vulnerability to the effect of chemotherapy and may therefore potentially affect cognitive function in the long-term survivors (Table 2). Correlative analysis was performed for all three groups between the SD_BOLD_ values and the three composite scores at TP1, between the SD_BOLD_ values at TP1 and score changes, and between SD_BOLD_ changes and score changes. A local FDR correction for multiple comparisons was performed in a small volume around each significant voxel in the size of a 5 × 5 × 5 neighborhood. The regional FDR-corrected statistical significance was set at q (p_FDR_) < 0.05.

## 3. Results

### 3.1. Study Participants

There were 20 participants in each of the three groups at TP1 (Table 1). At TP2, there were twelve remaining in the CH group, twelve in the NC group, and fifteen in the HC group. Reasons for attrition included the following: lost to follow-up, moved away, declined to continue, developed new cancer, having new memory problems, or deceased. Body mass index was the only parameter that was significantly different among the three groups (*p* = 0.004 for all participants and *p* = 0.002 for participants having data for both TP1 and TP2). At TP1, hormonal therapy was given to 29 cancer survivors, and there was no difference between the CH group and the NC group (*p* = 0.999). For survivors having data for both TP1 and TP2, hormonal therapy was given to 15 cancer survivors and there was no difference between the CH group and the NC group (*p* = 0.319). Regarding cancer staging, there was a significant difference between the CH group and the NC group at TP1 (*p* = 0.001). For survivors having data for both TP1 and TP2, there was a significant difference in cancer staging between the CH group and the NC group (*p* = 0.020).

### 3.2. SD_BOLD_ Data

**Figure 1:**
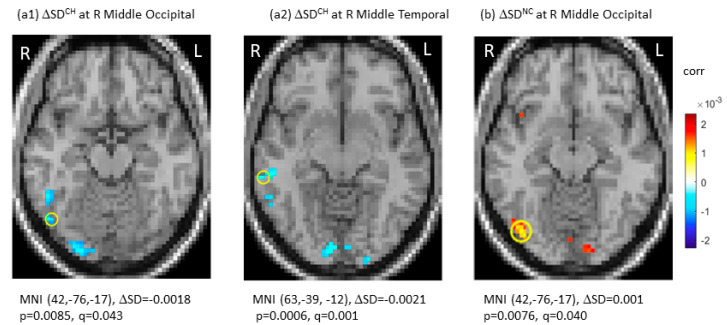
Significant longitudinal changes in blood-oxygen-level-dependent (BOLD) signal variability in standard deviation (ΔSD_BOLD_) in the chemotherapy group (CH) (**a1**,**a2**) and in the no-chemotherapy group (NC) (**b**). The significant regions were indicated with Montreal Neurological Institute (MNI) coordinates and marked by “o”. CH: chemotherapy group; FDR: false discovery rate; L: left side; NC: No-chemotherapy group; q = p_FDR_ after multiple comparison, R: right side, Δ: longitudinal change.

SD_BLOD_ data at TP1, the longitudinal changes (ΔSD_BLOD_), and the group-by-time interactions were presented in Table 2. Specifically, there were no significant differences in SD_BOLD_ among the three groups after multiple comparisons at TP1 (q (p_FDR_) > 0.05). Longitudinally, the CH group showed significant decreases in SD_BOLD_ in the right middle occipital gyrus (ΔSD_BOLD_ = −0.0018, *p* = 0.0085, q (p_FDR_) = 0.043 at MNI (42, −76, 17)) and right middle temporal gyrus (ΔSD_BOLD_ = −0.0021, *p* = 0.0006, q (p_FDR_) = 0.001 at MNI (63, −39, −12)). No significant SD_BOLD_ increases were noted in the CH group. For the NC group, there was a significant longitudinal SD_BOLD_ increase in the right middle occipital gyrus (ΔSD_BOLD_ = 0.0010, *p* = 0.0076, q (p_FDR_) = 0.040 at MNI (42, −76, −17)). For the HC group, there were no significant changes in SD_BOLD_ (q (p_FDR_) > 0.05). Figure 1 showed significant longitudinal changes (ΔSD) of SD_BOLD_ in the CH and NC groups under joint thresholding with |ΔSD_BOLD_| > 0.001 and q (p_FDR_) < 0.05.

**Figure 2:**
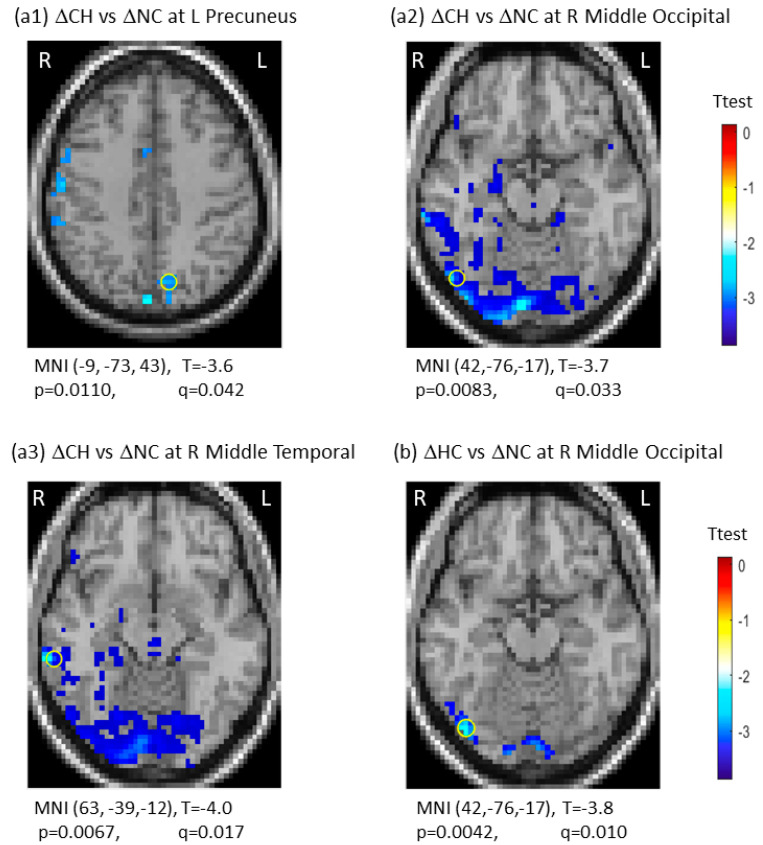
Significant group-by-time interactions of longitudinal changes in blood-oxygen-level-dependent (BOLD) signal variability in standard deviation (ΔSD_BOLD_) between the chemotherapy group (CH) and the no-chemotherapy group (NC) (**a1**–**a3**), and between the healthy control group (HC) and the no-chemotherapy group (NC) (**b**). The most significant regions were marked with “o”. CH: Chemotherapy group; FDR: false discovery rate; HC: healthy control group; L: left side; NC: No-chemotherapy group; q = p_FDR_ after multiple comparison, R: right side, Δ: longitudinal change.

There was group-by-time interaction between CH and NC groups in three regions, including the left precuneus (q (p_FDR)_ = 0.042), the right middle occipital gyrus (q (p_FDR_) = 0.033), and the right middle temporal gyrus (q (p_FDR_) = 0.017). For group-by-time interaction between HC and NC groups, we observed one significant region in the right middle occipital gyrus (q (p_FDR_) = 0.010). These results are presented in Figure 2. Note that the negative interaction between the CH and NC groups resulted from the ΔSD_BOLD_ (CH) decreases, indicating the CH group being less variable from TP1 to TP2 against the ΔSD_BOLD_ (NC) increases over time in the NC group. There were no significant group-by-time interactions for the CH versus the HC groups (Table 2). The 3D whole-brain SD_BOLD_ distributions at TP1 and TP2 are shown as a montage of axial slices in Figure S2. The 3D whole-brain longitudinal ΔSD_BOLD_ maps are presented in Figure S3.

### 3.3. Correlation between SD_BOLD_ and Neurocognitive Testing Scores

Neurocognitive testing showed a significant decrease in crystallized composite score (*p* = 0.04) and oral reading recognition (*p* = 0.02) in the CH group when compared to the NC group at TP1. Regarding the longitudinal changes, there were significant decreases in total composite score (*p* = 0.01), fluid composite score (*p* = 0.03), and picture vocabulary score (*p* = 0.04) in the CH group only. There were no significant longitudinal score changes in the NC or HC group. No group-by-time interactions were noted among the three groups.

**Figure 3:**
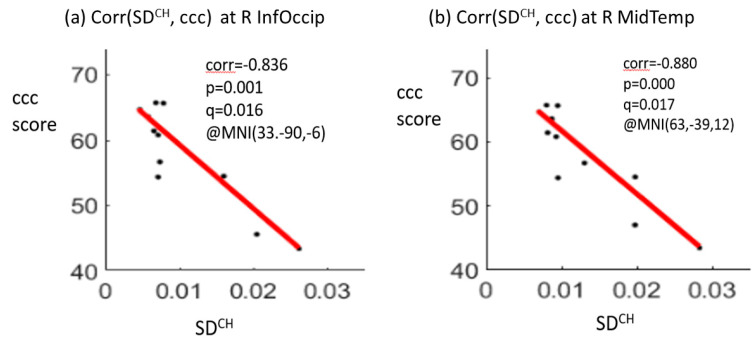
Significant negative correlations between the crystallized composite score (ccc) and the SD_BOLD_ (blood-oxygen-level-dependent signal variability in standard deviation) at the right inferior occipital gyrus (R InfOccip) (**a**) and right middle temporal gyrus (R MidTemp) (**b**) for the chemotherapy (CH) group at time point 1. Note: ccc, crystallized composite score; corr, correlation coefficient; *p*-value, t-test *p*-value; q-value, FDR-corrected *p*-value after multiple comparison; MNI (x, y, z), Montreal Neurological Institute coordinates (x, y, z); SD^CH^, SD_BOLD_ for the CH group.

The correlation analysis showed significant negative correlations between the crystallized composite scores and SD_BOLD_ values at two brain regions, including the right inferior occipital gyrus (correlation coefficient r = −0.836, *p* = 0.001, q (p_FDR_) = 0.016) and the right middle temporal gyrus (r = −0.880, *p* = 0.000, q (p_FDR_) = 0.017) for the CH group at TP1. A positive correlation between the total composite score and SD_BOLD_ value in the right middle temporal gyrus (r = 0.803, *p* = 0.003, q (p_FDR_) = 0.025) was noted for the NC group at TP1 (Figure 3). There were no significant correlations for the HC group at TP1. No significant correlations were noted between the longitudinal changes in the SD_BOLD_ of the two significant brain regions and the longitudinal changes in the composite scores for each of the three groups.

## 4. Discussion

In this study, we found diminished resting-state signal variability (decreased SD_BOLD_) in the posterior brain regions over a two-year interval in chemotherapy-treated older long-term survivors of breast cancer many years after chemotherapy. Signal variability and cognitive function were negatively correlated for the chemotherapy-treated group at the first assessment. To the best of our knowledge, this was the first longitudinal study of resting-state signal variability in older long-term survivors of breast cancer who were treated with chemotherapy.

Our findings of SD_BOLD_ decreases in the right middle occipital and right middle temporal gyri over time in the CH group were generally supported by prior literature on neuroimaging and CRCI. Prior fMRI studies by de Ruiter et al. revealed BOLD signal changes in the occipital and temporal cortex in long-term breast cancer survivors at ten years after chemotherapy [7,23]. In the study by McDonald et al., decreased gray matter density was noted at one month after chemotherapy and persisted for one year in several brain regions, including the temporal lobe. Their study implied continued structural alterations in chemotherapy-treated patients beyond the one-year interval [25]. Our own study of older women with a history of breast cancer identified greater reduction of gray matter density in the right middle temporal gyrus in the chemotherapy group as compared to the healthy control group [26]. Taken together, the occipital and temporal cortex may have alterations associated with chemotherapy treatment.

Our study identified the left precuneus as the region with significant interaction between the CH group and the NC group. Precuneus is a component of the DMN, a robust intrinsic functional brain network that supports implicit learning and cognitive processes [27,28]. Precuneus has been identified as a vulnerable brain region linked to CRCI. For example, Dumas et al. found a DMN reduction in the precuneus of patients with breast cancer at 1 month and 1 year after chemotherapy [29]. Our study of older women with breast cancer showed an acute alteration of intrinsic brain activity in the left precuneus shortly after chemotherapy [30].

We found no significant differences in SD_BOLD_ at the first assessment among the three groups, but significant changes in the CH group during the two-year interval. One speculation would rely on the possibility of the chemotherapy-treated group being partially recovered after treatment, and therefore no differences were detected at TP1. This was partly supported by a prior study performed by McDonald et al. [25], indicating partial recovery in brain gray matter density in bilateral superior frontal, right superior temporal, left middle frontal, and cerebellar regions at one-year post-chemotherapy. We would also speculate that the SD_BOLD_ signal decrease over time during the study period in the CH group was possibly due to the delayed effect of chemotherapy in our long-term survivors, which could be accelerated by aging since our study focused on older survivors at 65 years of age or older. However, we were limited by a small sample size for this study, which did not allow further analysis through stratification by age, i.e., the younger group (<75 years) and the older group (>75 years). Stratifying by age may help to identify possible SD_BOLD_ differences at TP1 and to observe how the younger group may be different than the older group. A future prospective longitudinal study with a large sample size of older survivors including pre-chemotherapy assessment and follow-up evaluations extending over five years is needed to identify the trajectory of SD_BOLD_ changes over time and its association with aging.

We used the BOLD signal variability in standard deviation, i.e., SD_BOLD,_ for this study because this parameter has been increasingly recognized as a neuroimaging marker of cognition and brain aging [15,16,17]. For instance, Garett et al. found that healthy adults with reduced BOLD variability over 2.5 years also had reduced cognition and functional integration, supporting a model wherein SD_BOLD_ was a measure of diminished cognitive functioning with aging [18]. Since their study validated the SD_BOLD_ as a marker of brain function over two years, we therefore used the same parameter for our study spanning over a two-year interval. In this study, we found longitudinal decreases in the composite scores from neurocognitive testing and a negative correlation between the composite scores and SD_BOLD_ in the CH group. The negative correlation in the CH group in our study contrasted with the prior studies of healthy adults showing a positive correlation between signal variability and cognitive testing scores, and a general trend of decreasing signal variability with aging [18]. However, our study also showed that the survivor group without chemotherapy had a positive correlation, which was similar to the prior studies in healthy adults [18]. Therefore, we speculate that other factors such as delayed detrimental effect of chemotherapy in addition to aging may play a role in the negative correlation observed in our chemotherapy-treated older survivors.

In this study, we assessed the potential neural correlates of cognitive function in older cancer survivors. However, the underlying mechanism of CRCI is still unknown [31]. A recent study reported the effect of chemotherapy on memory and concentration using positron emission tomography (PET)-MRI labeled with a radioligand of translocator protein (TSPO PET-MRI), which measured glial abundance [32]. They found higher TSPO expression in the parietal and occipital brain regions, which they attributed to neuroinflammation [32]. In the present study, we found decreased SD_BOLD_ in the posterior brain. Thus, it is reasonable to speculate that neuroinflammation may play a role in brain alterations, especially in the posterior brain regions of older long-term survivors.

There were several limitations. First, the sample size was small, and we experienced severe attrition over the two-year study interval. This attrition was partly attributed to medical and socioeconomic issues and to the requirement for MRI scans. Based on this experience, we will adopt a new strategy to minimize attrition for our future studies. Our strategies for enhancing participant retention and minimizing attrition include the following: engaging the participants to identify and troubleshoot issues/barriers; sending frequent reminders about their scheduled visits; helping them obtain social services and other resources such as bus passes, shuttle services, and medical transport if transportation is an issue; using cushions and support during the MRI scanning to alleviate discomfort and pain issues; and documenting reasons for participant withdrawal to improve retention for future studies. Second, with small samples, we did not have the statistical power to determine the effects of aging, cancer staging, chemotherapy regimen, and other clinical variables on brain function. The study was limited by variability in chemotherapy regimen, which was not properly controlled in the data analysis due to the small sample size. Nevertheless, the SD_BOLD_ data obtained from this study could be used to power a large study of aging and CRCI as SD_BOLD_ has been recognized as a neuroimaging marker of brain aging [15,16,17]. Our future study with a large sample size will assess a cohort stratified by age, such as the younger group (<75 years) and the older group (>75 years). Third, our correlative analysis only detected associations between SD_BOLD_ and neurocognitive testing scores at TP1 but not longitudinally. More studies need to be done to identify potential longitudinal neural correlates of CRCI in older long-term survivors of cancer.

In summary, we found diminished signal variability over two years in the posterior brain regions of older long-term survivors of breast cancer who were treated with chemotherapy many years ago. We also found a negative correlation between signal variability and cognitive function in the survivors who had chemotherapy. Our study supports the notion that SD_BOLD_ could be a potentially useful neuroimaging biomarker for late effects of chemotherapy on cognitive function in older long-term survivors of cancer.

## Figures and Tables

**Table 1:** Demographic and clinical information.

	All Participants				Participants Having Data for Both TP1 and TP2			
Parameters	CH N = 20	NC N = 20	HC N = 20	*p*	CH N = 12	NC N = 12	HC N = 15	*p*
Age (years)								
Mean (SD)	73.5 (5.06)	76.85 (4.63)	74.00 (6.09)	0.106	73.75 (5.41)	76.50 (4.28)	74.53 (6.73)	0.477
Median (Range)	73.5 (66–84)	77.5 (69–86)	72.5 (66–88)		71.50 (68–84)	75.5 (71–86)	73.00 (66–88)	
**Race** (N, %)								
White or Caucasian	15 (75)	18 (90)	18 (90)	0.100	10 (83)	11 (92)	14 (93)	0.765
Black	1 (5)	2 (10)			1 (8)	1 (8)		
Asian/Native Hawaiian	4 (20)		1 (5)		1 (8)		1 (7)	
Other			1 (5)					
**Ethnicity** (N, %)								
Not Hispanic	18 (90)	20 (100)	17 (85)	0.352	10 (83)	12 (100)	13 (87)	0.527
Hispanic	2 (10)		3 (15)		2 (17)		2 (13)	
**Marital Status** (N, %)								
Married/Partner	11 (55)	12 (60)	12 (60)	0.988	6 (50)	8 (67)	9 (60)	0.915
Not married	8 (40)	8 (40)	8 (40)		5 (42)	4 (33)	6 (40)	
Unknown	1 (5)				1 (8)			
**Highest grade** (N, %)								
High school or less	4 (20)	5 (25)	6 (30)	0.327	3 (25)	4 (33)	4 (27)	0.279
Some college	9 (45)	7 (35)	3 (15)		6 (50)	5 (42)	2 (13)	
Bachelor’s degree	5 (25)	7 (35)	6 (30)		2 (17)	3 (25)	5 (33)	
Advanced degree	2 (10)	1 (5)	5 (25)		1 (8)		4 (27)	
**Smoking** (N, %) ^*1^								
No	13 (65)	13 (65)	14 (70)	0.928	7 (58)	8 (67)	11 (73)	0.775
Yes	7 (35)	7 (35)	6 (30)		5 (42)	4 (33)	4 (27)	
**BMI** (kg/m^2^)								
Mean (SD)	30.78 (6.03)	27.11 (5.08)	24.83 (5.08)	0.004	29.89 (3.84)	27.01 (4.90)	23.63 (4.08)	0.002
Median (Range)	29.9 (22.4–43.8)	26.95 (18.7–35.9)	24.05 (16.6–37.5)		29.9 (23–37)	26.00 (21–36)	23.60 (17–31)	
**BOMC Score**								
Mean (SD)	2.90 (2.86)	3.05 (2.89)	2.89 (2.92)	0.982	3.33 (2.74)	1.83 (2.48)	2.71 (2.67)	0.385
Median (Range)	2 (0–8)	2 (0–10)	2 (0–10)		2 (0–8)	1(0–8)	2(0–8)	
**Stage** (N, %)								
DCIS	1 (5)	9 (45)			1 (8)	6 (50)		
I	4 (20)	8 (40)			1 (8)	4 (33)		
II	14 (70)	3 (15)			10 (84)	2 (17)		
III	1 (5)							
**Regimen** **Non-Trastuzumab Regimen** (N, %)								
AC-T	2 (10)							
TC	9 (45)				6 (50)			
AC	1 (5)				1 (8)			
CMF	1 (5)				1 (8)			
TAC	2 (10)				1 (8)			
Other ^*2^	1 (5)							
**Trastuzumab Regimen** (N, %)								
AC T + H	1 (5)				1 (8)			
TCH	1 (5)				1 (8)			
Other ^*3^	2 (10)				1 (8)			

^*1^ Lifetime cigarettes ≥ 100. ^*2^ Other included: Nab-paclitaxel, cyclophosphamide. ^*3^ Other included: Nab-paclitaxel, carboplatin, trastuzumab, doxorubicin and taxane. Abbreviations: AC, doxorubicin (brand name Adriamycin^®^) and cyclophosphamide; AC-T, doxorubicin and cyclophosphamide followed by paclitaxel (brand name Taxol^®^); AC T + H, doxorubicin and cyclophosphamide followed by paclitaxel and trastuzumab (brand name Herceptin^®^); BMI, body mass index; BOMC, Blessed Orientation-Memory-Concentration test; CH, breast cancer survivors exposed to chemotherapy; CMF, cyclophosphamide, methotrexate, and fluorouracil; DCIS, ductal carcinoma in situ; HC, healthy controls; NC, breast cancer survivors not exposed to chemotherapy; TAC, docetaxel (brand name Taxotere^®^), doxorubicin, and cyclophosphamide; TCH, docetaxel, carboplatin, and trastuzumab; TP1, time point 1; TP2, time point 2.

**Table 2:** Blood-oxygen-level-dependent signal variability in standard deviation (SD_BOLD_) data.

**1.SD_BOLD_ difference at timepoint 1 (TP1)** (thresholding by q < 0.05) * CH vs. HC:* None * CH vs. NC:* None * HC vs. NC:* None						
**2.Longitudinal SD_BOLD_ changes** (Δ**SD_BOLD_**): *CH:*						
Δ**SD_BOLD_**	t(*t*-test)	MNI	Region	*p*-value	q-value	
−0.0018	−4.0	(42, −76, 17)	Mid Occipital R	0.0085	0.043	
−0.0021	−4.6	(63, −39, −12)	Mid Temporal R	0.0006	0.001	
*NC:*						
Δ**SD_BOLD_**	t(t-test)	MNI	Region	*p*-value	q-value	
0.0010	3.9	(42, −76, −17)	Mid Occipital R	0.0076	0.040	
*HC:* None						
**3.Group-by-time interaction:** Δ**SD_BOLD_**						
*CH vs. HC:* None *CH vs. NC:*						
Δ**SD_BOLD_**(CH)	Δ**SD_BOLD_**(NC)	t(*t*-test)	MNI	Region	*p*-value	q-value
−0.0025	0.0010	−3.5	(−9, −73, 43)	Precuneus L	0.0110	0.042
−0.0019	0.0023	−3.7	(42, −76, −17)	Mid Occipital R	0.0083	0.033
−0.0013	0.0006	−4.0	(63, −39, −12)	Mid Temporal R	0.0067	0.017
* HC vs. NC:*						
Δ**SD_BOLD_**(HC)	Δ**SD_BOLD_**(NC)	t(*t*-test)	MNI	Region	*p*-value	q-value
−0.0012	0.0023	−3.8	(42, −76, −17)	Mid Occipital R	0.0042	0.010

Abbreviations: CH, breast cancer survivors exposed to chemotherapy; FDR, false discovery rate; HC, healthy controls; q-value, FDR-corrected *p*-value; Mid, middle; MNI, Montreal Neurological Institute; NC, breast cancer survivors not exposed to chemotherapy; R, right; SD_BOLD_, blood-oxygen-level-dependent signal variability in standard deviation; ΔSD_BOLD_, longitudinal change in SD_BOLD_.

## Data Availability

The data and materials will be available to researchers upon reasonable request.

## Supplementary Materials

The following are available online at https://www.mdpi.com/article/10.3390/brainsci12101283/s1, Figure S1: Representative blood-oxygen-level-dependent (BOLD) signal variability (SD_BOLD_) for differentiating brain functional signals captured at time point 1 (TP1) and time point 2 (TP2) in the posterior cingulum cortex. Note that the signals at the same voxel at TP1 (in black) and TP2 (in red) show spontaneous randomness of SD_BOLD_ but with a notable difference in signal fluctuations. Figure S2: Whole-brain three-dimensional distributions of blood-oxygen-level-dependent (BOLD) signal variability (SD_BOLD_) values at time point 1 (TP1) and time point 2 (TP2) for the breast cancer survivors exposed to chemotherapy (CH) (a1 and a2), the breast cancer survivors not exposed to chemotherapy (NC) (b1 and b2) and the healthy controls (HC) (c1 and c2) (display thresholding at SD_BOLD_ > 0.01). Figure S3: Whole-brain three-dimensional longitudinal changes of blood-oxygen-level-dependent (BOLD) signal variability (SD_BOLD_) values from time point 1 (TP1) to time point 2 (TP2) for the breast cancer survivors exposed to chemotherapy (CH). (a) Three-dimensional distributions of longitudinal SD_BOLD_ changes (display thresholding at ΔSD_BOLD_ > 0.002), (b) the statistical t-test map (thresholding at t-value > 2). The eligibility criteria checklists, and power analysis are also included in the supplementary materials.
