# Supplementary material for: Signal Variability and Cognitive Function in Older Long-Term Survivors of Breast Cancer with Exposure to Chemotherapy: A Prospective Longitudinal Resting-State fMRI Study
Source: Brain Sci. 2022 Sep 23;12(10):1283. doi: 10.3390/brainsci12101283 (PMC9599386; doi:10.3390/brainsci12101283)
Supplement: Supplementary file 1 [file brainsci-12-01283-s001.zip › brainsci-1913034-supplementary.pdf]

Supplementary files:

1. **Supplementary figures:**

Figure S1. Representative blood oxygenation level dependent (BOLD) signal variability (SD_{BOLD}) for differentiating brain functional signals captured at time point 1 (TP1) and time point 2 (TP2) in the posterior cingulum cortex. Note that the signals at the same voxel at TP1 (in black) and TP2 (in red) show spontaneous randomness of SD_{BOLD} but with a notable difference in signal fluctuations.

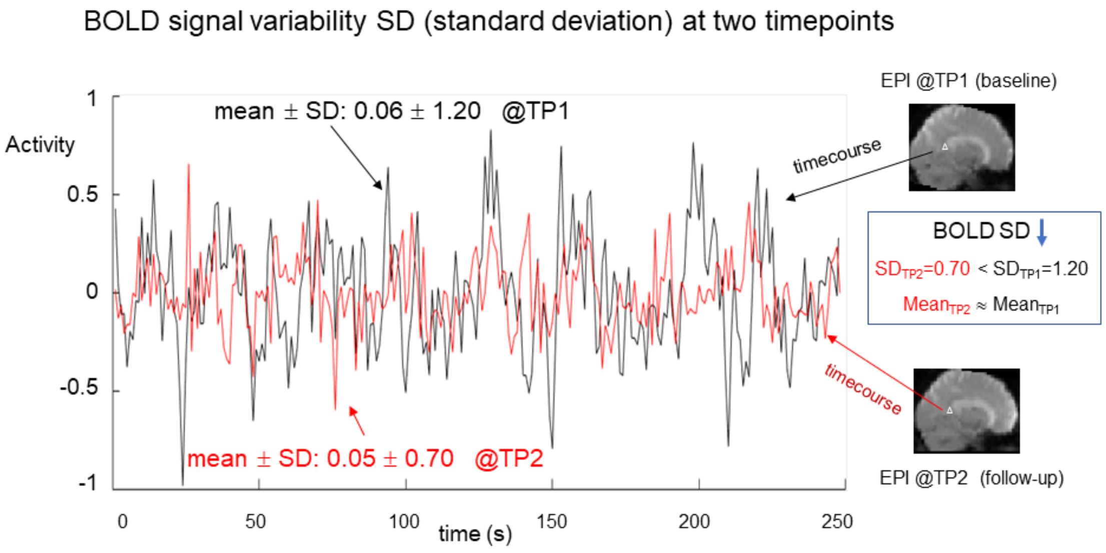

Figure S2. Whole-brain three-dimensional distributions of blood oxygenation level dependent (BOLD) signal variability (SD_{BOLD}) values at time point 1 (TP1) and time point 2 (TP2) for the breast cancer survivors exposed to chemotherapy (CH) (a1 & a2), the breast cancer survivors not exposed to chemotherapy (NC) (b1 & b2) and the healthy controls (HC) (c1 & c2) (display thresholding at $SD_{BOLD} > 0.01$).

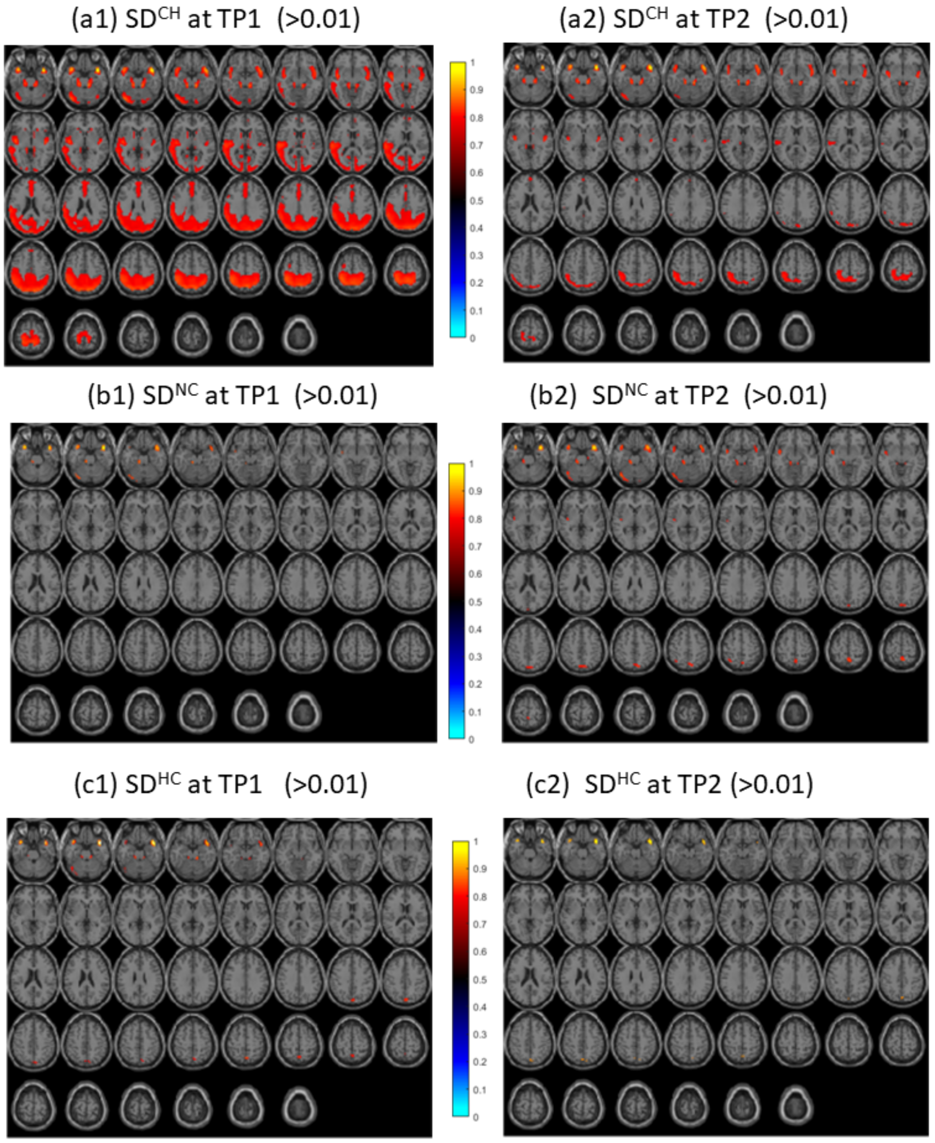

Figure S3. Whole-brain three-dimensional longitudinal changes of blood oxygenation level dependent (BOLD) signal variability (SD_{BOLD}) values from time point 1 (TP1) to time point 2 (TP2) for the breast cancer survivors exposed to chemotherapy (CH). (a) Three-dimensional distributions of longitudinal SD_{BOLD} changes (display thresholding at $\Delta SD_{BOLD} > 0.002$), and (b) the statistical t-test map (thresholding at T-value>2).

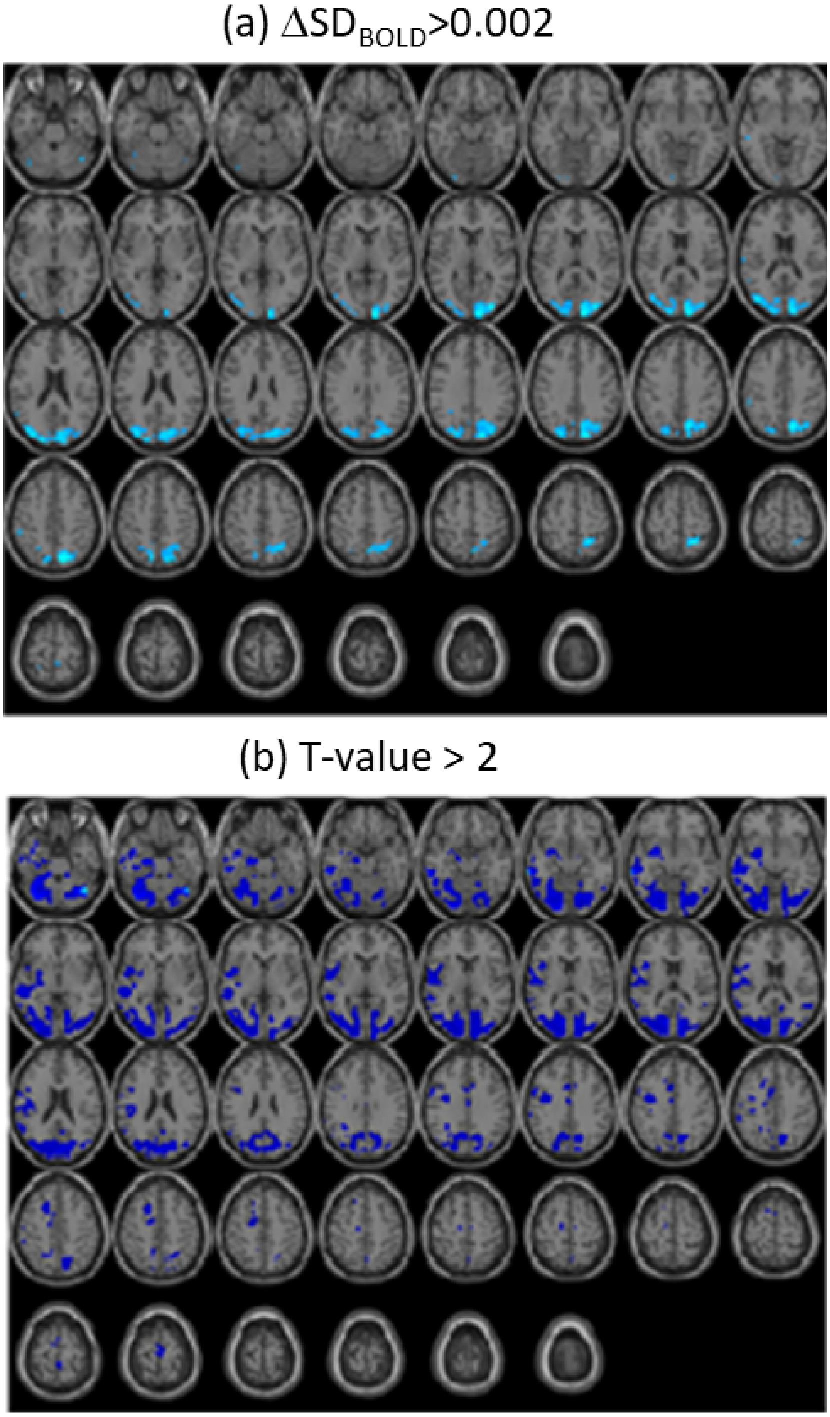

2. **Two eligibility criteria checklists used for this study:**

Eligibility Criteria	Yes	No
1. Are you taking anti-depressant or anti-anxiety medication?		
2. If you are taking anti-depressant or anti-anxiety medication, have you been taking the same medication and the same dose of the medication for at least 2 months?		
3. Since your last study visit, have you had a stroke or head injury requiring visit to the emergency room or hospitalization?		
4. Since your last study visit, have you had a diagnosis of cancer (except non-melanoma skin cancer)?		
5. Since your last study visit, have you received a diagnosis of a major Axis I psychiatric disorder including schizophrenia, manic-depressive disorder, or substance use disorder?		
6. Since your last study visit, have you received a diagnosis of a neurodegenerative disorder that affects cognitive function (e.g. Alzheimer's, Parkinson's, multiple sclerosis, dementia, seizure disorders, etc.)?		
7. Do you have any new visual or auditory impairment that would preclude ability to complete assessments?		

For Question 2: Answer must be YES
For Questions 3 – 7: Answers must be NO

Question	Yes	No
Is the participant eligible and enrolling onto IRB14063?	☐	☐
Is the person right handed?	☐	☐
Is the person claustrophobic or have a cardiac pacemaker or orbital metal implants, etc?	☐	☐

3. **Power analysis for this neuroimaging sub study:**

The power calculation for this neuroimaging sub study was based on the brain functional MRI (fMRI) reports from a published study by Kesler, et al, 2011. The dorsolateral prefrontal cortex (DLPFC) has been known as a region of the brain important for both executive function and working memory. Using the average fMRI contrast value over the Brodmann Area 10/46, a total sample of 20 patients with estimated contrast value between brain regions prior to chemotherapy of 3.7, and post-chemotherapy of 1.9, with an SD that was the minimum of the SD for between patient variation of 2.1 in the study by Kesler, et al, 2011, we would have 95% power to detect that difference in means, using paired t-test with a 0.05 two-sided significance level. No pre-specified multiple comparison adjustment would be conducted in the context of this exploratory study for the different cognitive tests considered.

Reference: Kesler, SR, Kent, JS, O'Hara, R. Prefrontal cortex and executive function impairments in primary breast cancer. Archives of Neurology 2011; 68:1447-53.
